# Building a Low-Threshold Model for HCV Diagnosis and Treatment Among Formerly Incarcerated Patients in Alabama

**DOI:** 10.1007/s11606-025-09411-y

**Published:** 2025-02-12

**Authors:** Margaret Hayden, Sanjay Kishore, Davis Bradford, Mikaela Dedona, Meghan Hunter, Mary Ellen Luck, Ryan Pratt

**Affiliations:** 1 University of Virginia Medical Center: UVA Health University Hospital, Charlottesville, VA USA; 2 Equal Justice Initiative, Montgomery, AL USA; 3 University of Alabama Birmingham Heersink School of Medicine, Birmingham, AL USA

**Keywords:** hepatitis C elimination, carceral health, health disparities, mobile clinic

## Abstract

**Background:**

Millions of Americans remain infected with hepatitis C (HCV). Innovation in care delivery is required to achieve the goal of national elimination.

**Aim:**

Develop a low-threshold HCV treatment program.

**Setting:**

Free clinic with mobile unit providing transitional care to people leaving jails and prisons across Alabama.

**Participants:**

Formerly incarcerated persons, many of whom are uninsured and live in rural areas.

**Program Description:**

We utilized point-of-care diagnostics to condense the HCV screening and pre-treatment evaluation into a single encounter. Patient assistance programs were used to obtain medications for uninsured patients. Clinical support was provided through in-person and telehealth care.

**Program Evaluation:**

From January 2023 to December 2024, 369 patients were screened for HCV; 104 (28.1%) were HCV antibody positive, and 71 (19.2%) were viremic. Of these patients, 70 completed pre-treatment diagnostics, 54 started treatment, 41 confirmed completion, 20 had SVR12 collected, with 19 achieving cure (94% cure rate). The median time from diagnosis to treatment initiation was 27 days.

**Discussion:**

It is possible to both diagnose HCV and complete the entire pre-treatment evaluation in a single encounter and initiate treatment within 1 month, even for predominantly uninsured populations in rural areas.

## INTRODUCTION

Hepatitis C virus (HCV) is one of the deadliest infectious diseases in America.^1^ The newest generation of direct acting antivirals (DAAs) are highly effective at curing all genotypes of HCV, can be prescribed by generalists in the vast majority of cases, and are well tolerated by patients.^2^ Despite this, less than 40% of all people diagnosed with HCV are started on medications within a year of diagnosis.^3^ The burden of disease is particularly severe for those who are incarcerated; estimates suggest between 12 and 35% of those in custody nationwide are infected with HCV.^4^ Lack of universal screening and financial barriers limit treatment rates for people while they are incarcerated; thus, programs that focus on improving access to care for those who have been recently released will be an important part of any hepatitis C elimination program.^5^ Given the competing demands of the re-entry time, which include healthcare but also finding stable housing, employment, navigating requirements of parole or other legal restrictions and re-integrating into relationships and communities, low-barrier healthcare interventions such as mobile clinics are essential. Mobile health interventions have shown promise in expanding access to HCV treatment in both rural and urban settings, although none to our knowledge have focused specifically on the formerly incarcerated.^6,7^

**Figure 1:**
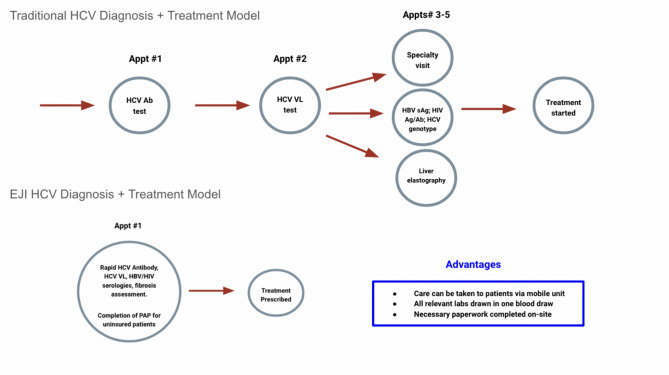
Graphic representation of traditional, multi-step HCV diagnostic and treatment algorithms as compared to EJI Health approach condensing evaluation to a single encounter with mobile outreach.

For all populations, traditional models of care have multiple points of friction for HCV evaluation (Fig. 1). Diagnosis of chronic HCV requires confirmation of a detectable viral load as roughly 25% of patients exposed to the virus (i.e., HCV antibody positive) will clear the infection without treatment.^8^ In addition, pre-treatment evaluation requires an assessment of liver fibrosis and additional testing to check for coinfection with hepatitis B and HIV.^2^ Furthermore, many payers require documentation of a genotype before covering medication, despite the availability of pangenotypic treatment options.^9^ An additional challenge for eradication of HCV over the past decade has been the exorbitant expense of medications, with first-line DAAs currently costing upwards of $25,000 per course.^10^

In order to address these challenges, the Equal Justice Initiative (EJI) developed and piloted a care delivery model that removed as many diagnostic barriers as feasible and consolidated the entire evaluation into a single encounter that was accessible both in a brick-and-mortar clinic and on a mobile clinic which visited parole offices. We also identified mechanisms for obtaining direct-acting antivirals for a group of individuals who were predominantly uninsured. Our goal was to demonstrate a successful model that could be replicated in other settings with high rates of hepatitis C and similar challenges (rural settings, high rates of poverty and uninsurance, and other social complexities).

## SETTING AND PARTICIPANTS

EJI is a non-profit legal organization dedicated to eliminating mass incarceration and addressing the legacy of slavery. For years, EJI has provided re-entry support to people leaving prison. Recognizing the need for expedited access to healthcare, EJI recruited a clinical team (two primary care physicians, one nurse, two clinic managers and patient navigators) to provide transitional primary care to its clients in 2022 and identified HCV as an initial area of focus. EJI developed a relationship with a network of parole centers and offered free screenings and basic primary care at a brick-and-mortar clinic in Montgomery, Alabama, as well as on site at parole centers across the state with a mobile clinic unit. In addition, EJI connected with local re-entry residential programs and substance use treatment facilities to offer opportunities for free HCV screening and treatment.

## PROGRAM DESCRIPTION

The EJI HCV program relies on point of care (POC) diagnostics, patient assistance programs from drug manufacturers, and dedicated staff time for patient navigation and support to diagnose patients, initiate treatment, and support patients through the process.

### Rapid Point of Care Diagnostics

To overcome the multi-step diagnostic process, EJI invested in POC laboratory equipment, relying on CLIA-waived equipment that could provide results within 15–20 min (OraQuick Hepatitis C Test, OraQuick Advance HIV-½ Rapid Antibody Test, Abaxis Piccolo Xpress with Complete Metabolic Panel reagent disc) and POC elastography (Echosens FibroscanGO). While the HCV and HIV antibody tests can be used with either fingerstick or venipuncture samples, our model relied on venipuncture to streamline follow-up and to allow us to run the complete metabolic panel (which requires venipuncture) and provide results to patients at the initial visit. Two serum-separator tubes were drawn on every patient: one tube was used to run samples for our point-of-care tests; the second tube was stored and, in the case of a positive HCV Antibody screen, was sent to an outside laboratory for evaluation of HCV viral load (and genotype if required by insurance), hepatitis B serologies, and HIV antigen/antibody assays. This approach minimizes needlesticks (single venipuncture, rather than fingerstick with follow-up venipuncture) and was acceptable to our patients. For those patients with a positive HCV antibody test, we also perform ultrasound elastography (e.g., FibroScan) during the initial evaluation to assess liver fibrosis. While fibrosis assessments can be performed using blood counts and chemistries (e.g., FIB-4, APRI scores), elastography is simple and effective and provides instantaneous results.^11^ Complete blood counts cannot be run in serum-separator tubes and would have complicated our streamlined phlebotomy procedures; hence, we relied on elastography. This equipment—POC blood tests and elastography—was utilized both in our brick and mortar clinic and on our mobile clinic. In either location, the entire diagnostic and pre-treatment evaluation could be condensed into a single visit with a single venipuncture.

### Treatment Initiation and Support

All patients with confirmed hepatitis C who met criteria for generalist treatment per AASLD/IDSA guidelines (i.e., no co-infection with HIV or hepatitis B, no decompensated cirrhosis) were offered treatment with either glecaprevir/pibrentasvir or sofosbuvir/velpatasvir.^2^ For those patients who were uninsured, the EJI Health clinic utilized patient assistance programs from drug manufacturers (Gilead and Abbvie). For patients with insurance, we dedicated staff time to navigating prior authorizations and co-pay assistance programs. Medication was shipped (directly from drug manufacturers or from specialty pharmacies, depending on a patient’s insurance status) to either our brick-and-mortar clinic in Montgomery or to patients’ homes depending on their preference and location. Once patients were started on treatment, we supported them via medication counseling, periodic phone calls during the course of treatment to encourage adherence, and visits at the 1-month mark to receive refills if they were local. Patients who did not have access to their cellphones often provided the number of the residence where they were staying (either with family or in a transitional supportive housing or “halfway house”). We attempted to confirm completion of the treatment course via telephone call and then arrange a blood draw at least 12 weeks following to confirm a cure (SVR12).

## PROGRAM EVALUATION

**Figure 2:**
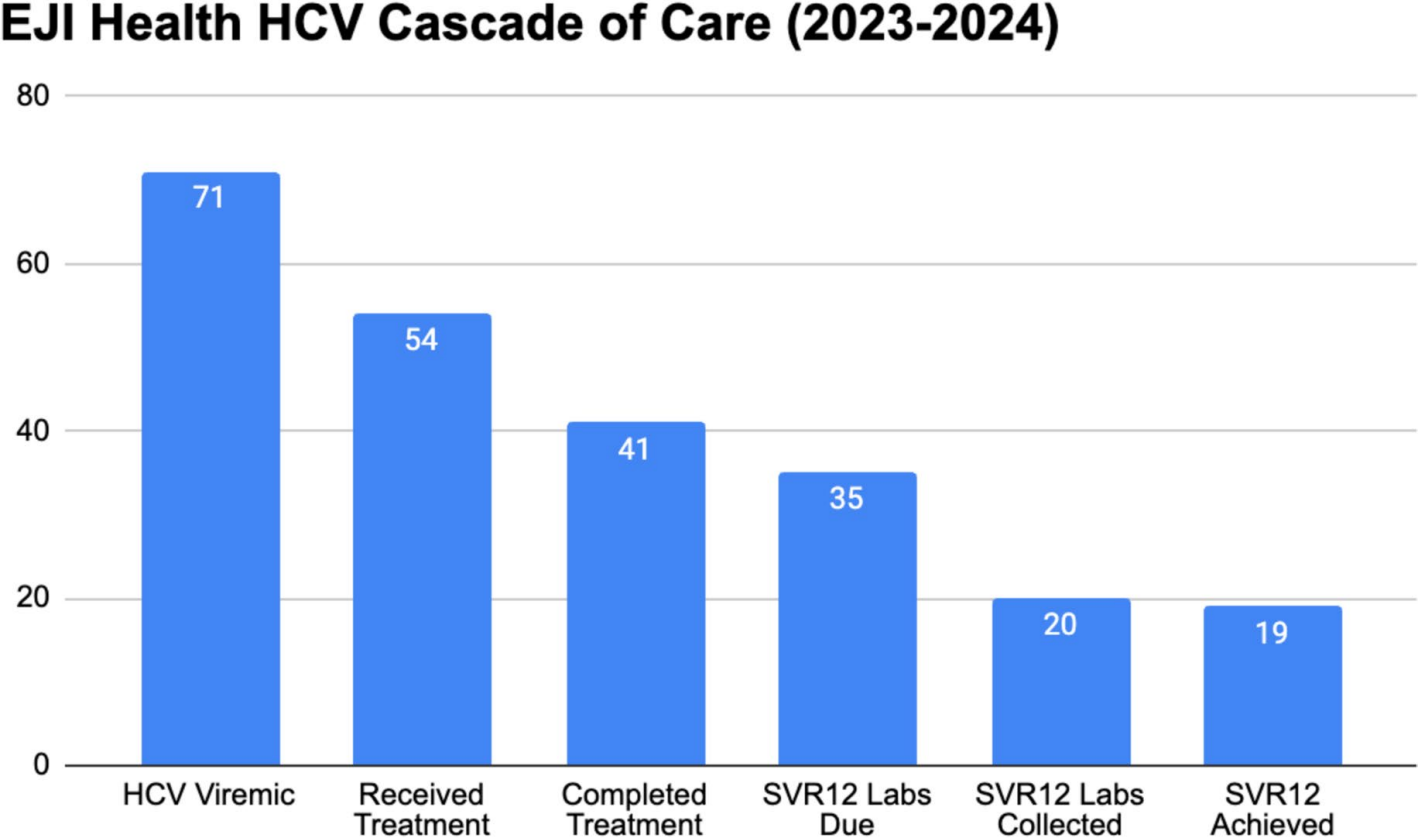
EJI Health Hepatitis C Cascade of Care from January 2023 to December 2024.

From January 2023 to December 2024, 369 patients were seen and screened for HCV. 104 (28.1%) were HCV antibody positive, and 71 (19.2%) were viremic. Of the 104 patients who were HCV antibody positive, 100 (96.1%) had an HCV viral load collected. Of the 71 patients who were viremic for hepatitis C, 100% received the initial diagnostic evaluation (assays for hepatitis B, HIV, and liver fibrosis). Of these patients, 54 started treatment, 41 were confirmed to complete treatment, 20 had SVR12 collected, with 19 achieving cure (95% cure rate) (Fig. 2). The viremic population was middle aged (mean age 43.8 years), predominantly identified as male (76%), White (85.9%), and uninsured (71.8%).

A key metric is time from diagnosis of HCV to treatment initiation; the median time from diagnosis to start of medication was 27 days (IQR 20–43.25 days). We find this varies based on insurance coverage, as some payers require burdensome prior authorization and limited coverage requiring negotiation with specialty pharmacies, co-pay assistance programs, and more. Uninsured patients were often able to get medications efficiently delivered via drug company patient assistance programs. As such, the median time from diagnosis to treatment initiation for uninsured patients was 25 days, compared to a median time of 35 days from diagnosis to start of medication for those patients with either public or private insurance. Treatment was delayed for medical reasons in three cases (new HIV diagnosis, new metastatic cancer diagnosis, and chronic hepatitis B infection).

Common obstacles to initiating treatment and confirming the completion of treatment included patients losing reliable access to their phones, difficulty obtaining financial information required by drug companies for patient assistance programs, and reincarceration.

## DISCUSSION

This pilot program demonstrates that it is possible to condense the entire hepatitis C treatment evaluation into a single encounter. In traditional models, each subsequent step—e.g., confirmation of viral load, assessment of comorbidities, liver fibrosis assessment, and treatment initiation—presents an opportunity for patients to become lost to follow-up. In addition, we show that among formerly incarcerated patients, even in states without Medicaid expansion, it is possible to treat hepatitis C. Our high cure rate among those who had a SVR12 blood draw is in agreement with previous studies that show very high cure rates in populations with substance use disorders and those without perfect adherence. This is further evidence that low-threshold, easy-access HCV treatment programs are key to getting lifesaving medication to the patients who need it.

Our program was successful in part because of philanthropic donations to support the cost of staff, supplies, and diagnostics. Moreover, we are dependent on the charity of manufacturers (via patient assistance programs) for therapeutics for all uninsured patients. Given Alabama’s failure to expand Medicaid, in order to make programs like ours sustainable and to move the needle on hepatitis C elimination, there will need to be both large national investments in funding for therapeutics and diagnostics and policy advocacy to ease various restrictions that limit access to these lifesaving medications. The National Institutes of Health is proposing a plan to invest over $5 billion to overcome barriers of cost and limited diagnostics to improve access to treatment with the goal of eliminating HCV, and a new point of care HCV viral load assay was recently approved by the FDA.^12^ These are exciting developments and will hopefully build momentum for additional policy changes necessary to improve the hepatitis C treatment landscape. The new POC viral load assay is promising but to move to a model that would allow same day testing, diagnosis, and treatment initiation, point of care hepatitis B serum antigen testing (which is approved in Europe) will also need to be approved in the USA. Mobile pharmacies and other financing innovations that would allow for the provision of medications directly at the time of diagnosis will also be important.

Due to the criminalization of substance use, relapses among our patients often led to incarceration which interrupted both substance use disorder treatment and their hepatitis C treatment. In addition, options for standard of care addiction medicine such buprenorphine/naloxone for opioid use disorder or contingency management for stimulant use disorder remain few and far between. Promoting treatment of substance use disorders over criminalization and increasing access to addiction medicine are key to the long-term success of any HCV elimination project. At a payer level, formulary restrictions greatly delay treatment initiation. These vary across insurance programs and can include fibrosis restrictions (e.g., requiring F2 or greater fibrosis), prescriber restrictions (e.g., requiring a ID or GI specialist to be involved in care), substance use related (e.g., requiring a certain amount of sobriety or involvement in a treatment program), or viral related (e.g., requiring a genotype). None of these restrictions are supported by evidence and create additional layers of bureaucracy which inevitably cause a drop off in care; indeed, evidence suggests that when state Medicaid programs relax coverage restrictions, antiviral prescriptions increase.^13^

Hepatitis C represents the potential of a true public health moonshot: with the newest generation of antivirals, elimination of this deadly virus is possible. This ambitious goal will require innovative approaches to bring state of art medicine to those who need it most. This pilot project demonstrates that even in rural areas without Medicaid expansion, it is possible to diagnose and cure patients with hepatitis C.

## Data Availability

The initial program data analyzed during this report are available from the corresponding author on reasonable request.

## References

[1] **Ly KN, Hughes EM, Jiles RB, et al.** Rising Mortality Associated With Hepatitis C Virus in the United States, 2003–2013. Clin Infect Dis. 2016; 62(10): 1287-8. 10.1093/cid/ciw111. 26936668 10.1093/cid/ciw111 PMC11089523

[2] AASLD-IDSA Hepatitis C Panel, Hepatitis C guidance 2019 update: AASLD-IDSA recommendations for testing, managing, and treating hepatitis C virus infection. Hepatology. 2020; 71(2): 686–721. https://www.ncbi.nlm.nih.gov/pmc/articles/PMC9710295/. Accessed 2/6/2025. 10.1002/hep.31060 PMC9710295 31816111

[3] **Thompson WW. Symum H, Sandul A, et al.** Vital signs: hepatitis C treatment among insured adults—United States, 2019–2020. Morbidity and Mortality Weekly Report 2022; 71(32): 1011–1017. https://www.ncbi.nlm.nih.gov/pmc/articles/PMC9400534/. Accessed 2/6/2025. 10.15585/mmwr.mm7132e1 PMC9400534 35951484

[4] **Florko N.** Hundreds of incarcerated people are dying of hep c - even though we have a simple cure. Stat News. December 15, 2022. Accessed March 5, 2024. https://www.statnews.com/2022/12/15/hundreds-incarcerated-people-dying-hepatitis-c-despite-simple-cure/. Accessed 2/6/2025.

[5] **McNamara M, Furukawa J, Cartwright EJ.** Advancing hepatitis C elimination through opt-out universal screening and treatment in carceral settings, United States. Emerg Infect Dis. 2024; 30 (Suppl 1): S80. 38561831 10.3201/eid3013.230859 PMC10986823

[6] **Rosencrans A, Harris R, Clair S, et al.** Challenges and Opportunities for Treating Hepatitis C Amongst People Who Use Drugs: Experience of an Integrated Mobile Clinic in Baltimore City. J Viral Hepat. 2024. 10.1111/jvh.14038. 10.1111/jvh.14038 PMC13249361 39588790

[7] **Rennert L, Howard KA, Kickham CM, et al.** Implementation of a mobile health clinic framework for hepatitis C virus screening and treatment: a descriptive study. The Lancet Regional Health–Americas. 2024;29:100648. 10.1016/j.lana.2023.100648. 10.1016/j.lana.2023.100648 PMC10733089 38124995

[8] **Micallef J, Kaldor JM, Dore GJ.** Spontaneous viral clearance following acute hepatitis C infection: a systematic review of longitudinal studies. J Viral Hepat. 2006; 13(1): 34–41. https://pubmed.ncbi.nlm.nih.gov/16364080/. Accessed 2/6/2025. 10.1111/j.1365-2893.2005.00651.x 16364080

[9] Center for Health Law and Policy Innovation & National Viral Hepatitis Roundtable, Hepatitis C: State of Medicaid Access (2024). www.stateofhepc.org. Accessed 2/6/2025

[10] **Barber MJ, Gotham D, Khwairakpam G, et al.** Price of a hepatitis C cure: Cost of production and current prices for direct-acting antivirals in 50 countries. J Virus Eradication. 2020;6(3), 100001. https://www.sciencedirect.com/science/article/pii/S2055664020300017. Accessed 2/6/2025. 10.1016/j.jve.2020.06.001 PMC7646676 33251019

[11] **Castéra L, Vergniol J, Foucher J et al.** Prospective comparison of transient elastography, Fibrotest, APRI, and liver biopsy for the assessment of fibrosis in chronic hepatitis C. Gastroenterology. 2005;128(2):343-50. 10.1053/j.gastro.2004.11.018. 15685546 10.1053/j.gastro.2004.11.018

[12] **Fleurence R, Collins F.** A National Hepatitis C Elimination Program in the United States. JAMA. 2023;329(15):1251-1252. 10.1001/jama.2023.3692. 36892976 10.1001/jama.2023.3692

[13] **Davey S, et al.** Changes in Use of Hepatitis C Direct-Acting Antivirals After Access Restrictions Were Eased by State Medicaid Programs. JAMA Health Forum. 2024;5(4):e240302. 10.1001/jamahealthforum.2024.0302. 38578628 10.1001/jamahealthforum.2024.0302 PMC10998155

